# Fungicide-Loaded Liposomes for the Treatment of Fungal Diseases in Agriculture: An Assessment of *Botrytis cinerea*

**DOI:** 10.3390/ijms25158359

**Published:** 2024-07-31

**Authors:** Angelo Agnusdei, Anna Maria Maurelli, Donato Gerin, Donato Monopoli, Stefania Pollastro, Lucia Catucci, Francesco Faretra, Vincenzo De Leo

**Affiliations:** 1 Department of Soil, Plant and Food Sciences, University of Bari Aldo Moro, Via Amendola 165/A, 70126 Bari, Italy; angelo.agnusdei@uniba.it (A.A.); donato.gerin@uniba.it (D.G.); francesco.faretra@uniba.it (F.F.); 2 Department of Chemistry, University of Bari Aldo Moro, Via Orabona 4, 70126 Bari, Italy; anna.maurelli@uniba.it (A.M.M.); d.monopoli2@alumni.uniba.it (D.M.); vincenzo.deleo@uniba.it (V.D.L.)

**Keywords:** liposome, fungicide, Fludioxonil, agriculture, fungal diseases, *Botrytis cinerea*, conidial germination test, germ tube elongation, colony radial growth

## Abstract

In this work, liposomes loaded with the fungicide, Fludioxonil (FLUD), for the containment of fungal diseases in agriculture were developed. Three types of vesicles with different compositions were compared: (I) plain vesicles, composed of soy phosphatidylcholine and cholesterol; (II) PEG-coated vesicles, with an additional polyethylene glycol coating; and (III) cationic vesicles, containing didodecyldimethylammonium bromide. Nanometric-sized vesicles were obtained both by the micelle-to-vesicle transition method and by the extrusion technique, and encapsulation efficiency, drug loading content, and Zeta potential were determined for all the samples. The extruded and PEGylated liposomes were the most stable over time and together with the cationic ones showed a significant prolonged FLUD release capacity. The liposomes’ biological activity was evaluated on conidial germination, germ tube elongation and colony radial growth of the ascomycete *Botrytis cinerea*, a phytopathogenic fungus affecting worldwide many important agricultural crops in the field as well as in the postharvest phase. The extruded and PEGylated liposomes showed greater effectiveness in inhibiting germ tube elongation and colony radial growth of the fungal pathogen, even at 0.01 µg·mL^−1^, the lowest concentration assessed.

## 1. Introduction

To address the challenges of more efficient and sustainable agriculture, in recent years, nanotechnology has been applied in agronomic practices not only to increase crop productivity and quality but also in the postharvest phase to improve shelf life and reduce product losses [1]. The most consolidated applications of nanotechnology in agriculture concern the use of nanomaterials to reduce the application rate of herbicides, minimize nutrient losses during fertilization and increase production through pest and nutrient management [1,2,3,4]. Several nanofertilizers and nanopesticides have been proposed by researchers and tested at lab or greenhouse scale, and some nanofertilizers have been developed as commercial products [5,6]. Nanoparticles can be used in two ways to protect plants: either (a) the nanoparticles themselves can exert a protective or ameliorative function on crops, or (b) they act as carriers of already existing active substances [7]. The latter case is mainly applied to the delivery of poorly water-soluble compounds and to obtain stable formulations with enhanced bioavailability and efficacy. This also results in reduced dispersion in the environment and therefore a lower impact on the ecosystem by often problematic molecules [8]. For this purpose, nanocarriers for the development of nanofungicides, nanobactericides and nanoinsecticides have been proposed in the last decade [5]. For example, xylan- and lignin-based nanocarriers were used to encapsulate fungicides (such as pyraclostrobin, azoxystrobin, tebuconazole, and boscalid), and their in vitro activity was successfully tested against several pathogenic fungi (*Phaeomoniella chlamydospora*, *Phaeoacremonium minimum*, etc.), which are responsible for fungal plant diseases [9,10,11].

To prepare the nanostructured carriers, researchers have often drawn on materials and assemblies already developed for applications in the biomedical field: inorganic porous nanoparticles, polymer-based nanoparticles, surfactants for nanoemulsions, and lipids organized into nanoparticles and liposomes [12]. These systems are generally of proven environmental and human health safety and are therefore useful for mitigating the public concern about the health risks associated with nanotechnology-based products.

Liposomes are supramolecular assemblies of amphiphilic building blocks, usually phospholipids, forming bilayer structures organized in closed, spherical vesicles dispersed in an aqueous solution [13]. Liposomes can be easily prepared and adapted to the most disparate experimental and application needs by modulating their composition and surface characteristics [14]. Although liposomes can encapsulate both hydrophilic and hydrophobic compounds [15], their greatest application potential in agriculture concerns the possibility of conferring high colloidal stability and efficient delivery to poorly water-soluble compounds, thus ensuring their improved bioavailability. Furthermore, it has been also demonstrated that liposome-based nanocarriers can penetrate the leaves and be internalized by the plant cells, resulting in a high-precision target site release of the active compounds [16].

Building blocks of natural origin are often used to produce liposomes, such as phosphatidylcholine (PC), one of the main components of biological membranes, which can be easily extracted from a variety of natural sources such as egg yolk or soybeans. The obtained carriers are fully biocompatible and biodegradable, without problems of dispersion and persistence in the environment [14].

Ascomycota *Botrytis cinerea* is a ubiquitous fungal plant pathogen responsible for grey mold, a disease causing significant yield losses in a wide range of crops under different climatic conditions, and it is reported as the most important postharvest pathogen [17,18].

Different control strategies are used to manage *B. cinerea* infection, but chemicals remain the most solid tools for controlling the pathogen, particularly in the presence of high disease pressure [19]. The fungicide Fludioxonil (FLUD), a phenylpyrrole derived from pyrrolnitrin, an antibiotic produced by the bacterium *Pseudomonas pyrrocinia* and other species of the genus *Pseudomonas*, strongly affects conidial germination and mycelial growth [20,21].

However, the onset of multidrug resistance mechanisms [22], polyphagia and the capability to use several pathogenesis mechanisms [23], as well as increasing public concern over the negative side effects of chemicals on the environment and human health, require the development of new and more effective control strategies against *B. cinerea*, including the improvement of the drug delivery to reduce extensive spraying of agrochemicals.

In this work, FLUD-loaded liposomes for the containment of fungal diseases in agriculture were developed. Two methods were compared for the preparation of liposomes: the micelle-to-vesicle transition (MVT) method and the extrusion method. Furthermore, three types of vesicles different in composition were compared: (I) plain vesicles, (II) PEGylated vesicles, and (III) cationic vesicles. The resulting FLUD-loaded liposomes were characterized in terms of size, encapsulation efficiency (EE%), drug loading content (DLC%), surface charge, stability, and release performances. In vitro experiments were then carried out to evaluate the release behavior of liposomes and their biological activity against *B. cinerea*.

## 2. Results and Discussion

### 2.1. Liposome Preparation and Characterization

**Scheme 1:**
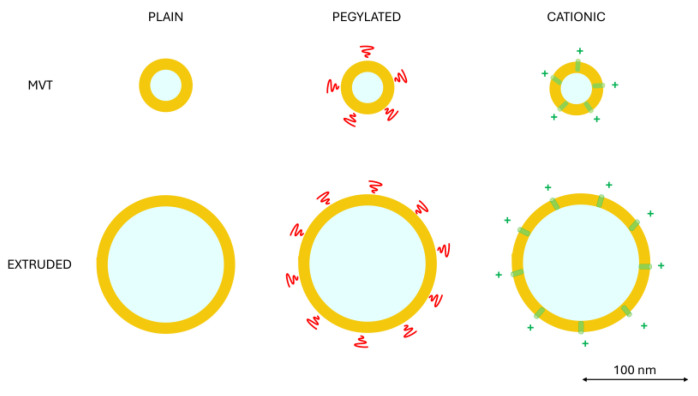
Schematic representation of the types of liposomes used for the delivery of FLUD to *Botrytis cinerea*. Only the diameters are to scale.

The size of liposomes affects their biological activity since vesicles of different sizes can give rise to different cellular uptake mechanisms, and ultimately different concentrations and localizations of their payload within the microbial target. Therefore, in this work two preparation methods were evaluated, i.e., the MVT method and the extrusion method, to obtain liposomes of different average dimensions, but both in the nanometric range. Also, the surface characteristics of liposomes, such as the charge or the presence of polymeric coatings, greatly influence their biological behavior. Therefore, for each of the two preparation methods used, three liposomal formulations were prepared to obtain a total of six types of FLUD-loaded nanoliposomes to be tested against *B. cinerea* (see Scheme 1). Cholesterol was added to all the liposome formulations because it increases the mechanical rigidity of the lipid bilayer in the headgroups region, increasing the colloidal stability of the nanocarriers. Furthermore, cholesterol, intercalating in the lipid palisade, decreases the lipid bilayer packing defects and causes an increase in the incorporation efficiency of hydrophobic molecules [24]. To obtain more colloidally stable liposomes, a PEG coating was introduced into one of the formulations, which offers a steric barrier to the vesicles, preventing their aggregation. Finally, cationic vesicles containing DDAB were considered, since positive charges on liposome surfaces can improve the interaction between nanocarriers and negatively charged surfaces of microbial cells [25].

**Figure 1:**
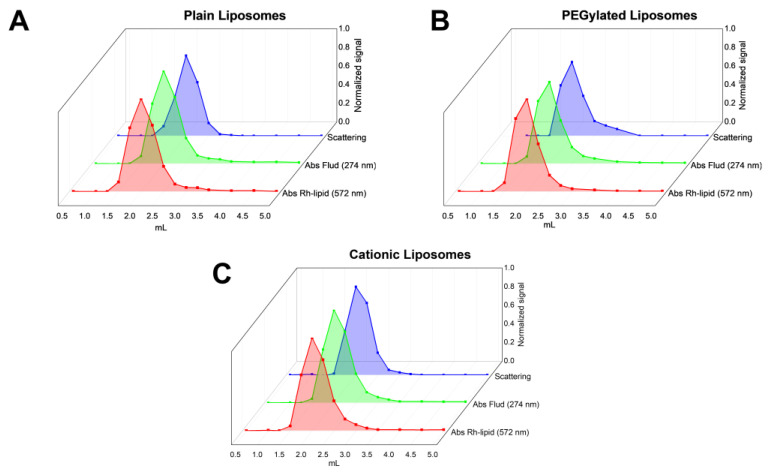
Elution profile on Sephadex G50 medium of plain liposomes (**A**), PEGylated liposomes (**B**) and cationic liposomes (**C**) loaded with FLUD (lipids: mg·mL^−1^; FLUD: 30 µg·mL^−1^).

The MVT method, compared to other liposome preparation techniques, allows for obtaining homogeneous populations of unilamellar vesicles of small size in a brief time (less than five minutes). The method is based on the removal of detergent molecules by size exclusion chromatography (SEC) from mixed phospholipid/detergent micelles to induce the formation of liposomes (see Section 3.2). Since the quality of the produced liposomes and the EE% depend on the efficiency of the SEC, preliminary experiments were conducted to determine the maximum quantity of lipids to be used for each batch of liposomes to avoid column overloads and to set the optimal lipid/FLUD ratio for maximum encapsulation performance. The elution profiles obtained by means of spectroscopic and Dynamic Light Scattering (DLS) measurements relevant to the best formulations (8 mg·mL^−1^ lipid concentration; 83.3 lipids/FLUD molar ratio) are shown in Figure 1. For each typology of liposomes tested, Figure 1 shows that the FLUD molecules eluted together with those of the lipids (for this purpose, lipids absorbing in the visible region were included in the formulation). In addition, both curves were superimposable to the profile obtained by monitoring light scattering during elution, which leads to the conclusion that the FLUD and lipids coeluted in the form of vesicles. A 1 mL fraction was collected after 1.5 mL of dead volume for each elution, as previous studies have shown that beyond this limit it is possible to incur contamination from minimal quantities of detergent [26].

Table 1 shows the characteristics of the liposomes obtained by the MVT method. The average dimensions measured were in the range of 30–60 nm and the EE% values were just under 100%. The maximum DLC% value of 0.38% was recorded for the unmodified liposomes (plain), but the other samples also reached similar values. The Z potential was slightly negative for the plain sample, as already reported for vesicles composed of PC and cholesterol [27], and attenuated slightly following coating with PEG. The addition of DDAB to the cationic sample formulation resulted in a Z potential value of approximately +18.2 mV.

The extrusion method involves the resizing of the lipid vesicles obtained by natural swelling by passage through polycarbonate membranes with defined porosity. In this work, membranes with a porosity of 100 nm were used, and the method was applied to the lipid concentration and lipid/FLUD ratio identical to those set for the MVT method. Table 1 shows that the parameters recorded for the vesicles obtained with this method were in line with those previously obtained, except for the average dimensions, which were within the range of 120–130 nm.

### 2.2. Liposome Stability

**Figure 2:**
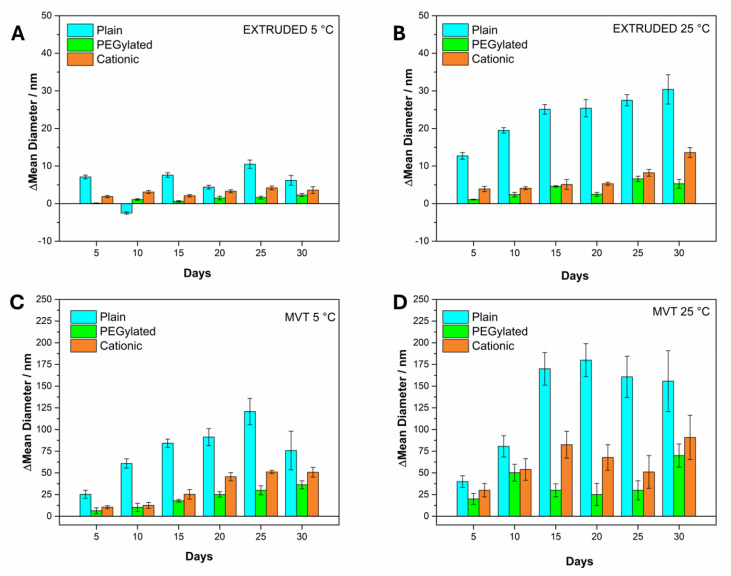
Stability over time of plain, PEGylated and cationic liposomes, in terms of Δ (mean diameter). (**A**) Stability of extruded samples at 5 °C and (**B**) 25 °C. (**C**) Stability of samples obtained by MVT method at 5 °C and (**D**) 25 °C. Data are expressed as mean values ± standard deviation (*n* = 3).

The colloidal stability of the six prepared liposomes was evaluated in vitro by monitoring the variations in particle size with respect to the initial values over time, keeping the samples at 5 °C and 25 °C (Figure 2). As expected, all the samples stored at low temperatures (Figure 2A,C) showed higher stability than those stored at 25 °C (Figure 2B,D). In fact, it is known that the fluidity of the liposome membranes increases with the rise in temperature, affecting their stability [28].

The comparison between the vesicles prepared by extrusion (Figure 2A,B) and with the MVT method (Figure 2C,D) shows how, in general, the latter have undergone greater variations in diameter over time. The liposomes made with the MVT method had a very small starting average size (between approximately 30 and 60 nm) and therefore a higher curvature of lipid membranes than the larger liposomes made by extrusion. Higher curvatures correspond to a greater number of packing defects in the lipid bilayer, with a consequent decrease in the stability of liposomes [29,30].

Considering both preparation methods and storage conditions tested, the largest variations were found in the plain liposomes, especially at room temperature. These vesicles were made without any stabilizing building blocks and were characterized by a weakly negative Z potential and therefore not capable of ensuring electrostatic stabilization. On the contrary, the PEGylated liposomes were the most stable in both conditions tested, due to the steric stabilization provided by the presence of their polymeric coating. These vesicles were obtained by adding 7% mol 1,2-distearoyl-sn-glycero-3-phosphoethanolamine-N-[carboxy(polyethylene glycol)-2000] (sodium salt) (DSPE PEG-2000) to the lipid formulation, which ensures a full PEG coating in “brush” conformation, capable of limiting the fusion between the vesicles by introducing a physical barrier on the liposomal surface [31]. Moderate variations in the average size were also recorded for the cationic liposomes, as the positive charge introduced on the vesicle surface by the DDAB molecules ensured a certain electrostatic stabilization [32].

### 2.3. Release Behaviors of FLUD-Loaded Liposomes

**Figure 3:**
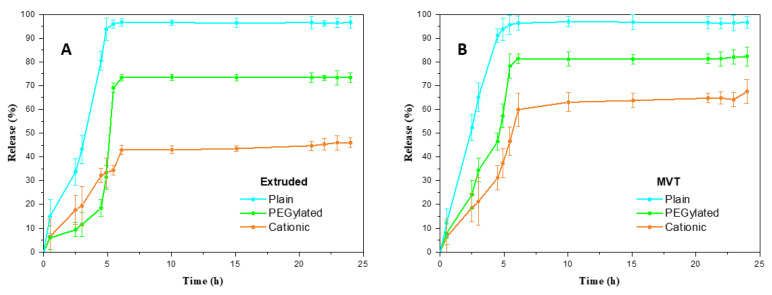
Release behaviors of FLUD-loaded liposomes for 24 h. (**A**) Liposomes obtained by extrusion; (**B**) liposomes obtained by the MVT method. Data are expressed as mean values ± standard deviation (*n* = 3).

The FLUD release profiles from the liposomes obtained through the extrusion and MVT methods are shown in Figure 3A and Figure 3B, respectively. All the types of liposomes showed a significant prolonged release capacity in the first six hours, albeit with decreasing speeds going from plain to cationic samples. Interestingly, this finding is similar to that previously observed for the fungicide Cymoxanil released from non-phospholipid liposomes [33]. In this time frame, all the liposome samples reached a situation of substantial equilibrium, and no further release phases were observed. Except for the plain samples, which at equilibrium released approximately 95% of the FLUD, the other liposomes reached significantly lower release values, approximately between 70% and 80% for the PEGylated samples and between 45% and 65% for the cationic samples. The lipid composition ultimately appears to have influenced both the initial FLUD release rate and the maximum release value at equilibrium. Compared to plain liposomes, the PEG polymer coating could conceivably behave as a barrier that reduces the diffusion of FLUD from the lipid bilayer toward the outside of the PEGylated carriers. Instead, in the cationic samples, the tighter molecular packing that occurs between the constituent elements of the lipid bilayer could be the cause of the observed reduction in the drug release rate. Previous studies have shown that electrostatic interactions between cationic DDAB and zwitterionic PC lipids lead to a reorientation of the head groups of PC molecules and a stronger hydrophobic interaction between the hydrocarbon chains in the phospholipid bilayer [34]. The liposomes obtained with the MVT method show, for the PEGylated and cationic liposomes, release values at equilibrium that are, on average, larger than the liposomes obtained by extrusion. This effect could be due to the smaller size of these samples and the higher surface area, which resulted in more effective exchange conditions between the liposomes and the receiving medium used for the assay. Even the greater initial rates of FLUD release observed for liposomes made using the MVT method are related to the greater degree of curvature and therefore packing defects of the bilayers of these vesicles that, in addition to stability, also affect the release properties of their payload, facilitating it [29,30].

### 2.4. Antifungal Activity

Interfering with cellular osmoregulation mechanisms, Fludioxonil can exert a containment action both in the early stages of conidial germination and in the subsequent phases of germ tube elongation and radial growth of the colony. For this reason, the antifungal activity of the FLUD-loaded liposome formulations was evaluated in vitro against *B. cinerea*, reference strain SAS56 (CBS 145097), in terms of conidial germination, germ tube elongation and colony radial growth.

The data and statistical information on the antifungal effectiveness on conidial germination and germ tube elongation for both methods are reported in Table 2.

No antifungal effects were observed using unloaded liposomes in all the tested proposed formulations.

**Figure 4:**
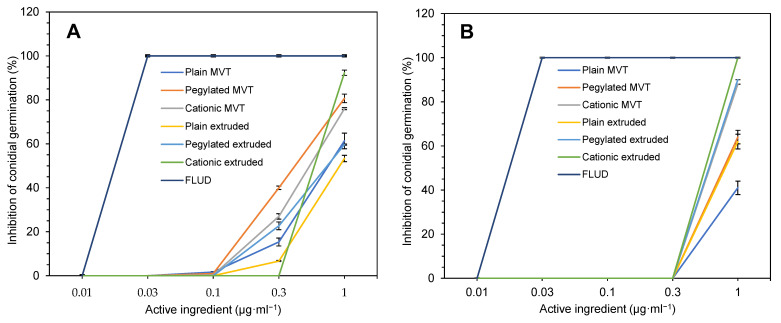
Inhibition of *B. cinerea* conidial germination following the application of different doses of liposome formulations or FLUD. (**A**) FLUD and FLUD-loaded liposomes were incorporated into the growth medium (method 1). (**B**) FLUD and FLUD-loaded liposomes were applied on the top of the medium (method 2). Bars indicate the standard error of three biological replicates.

The known minimum inhibitory concentration (MIC), 0.03 µg·mL^−1^, was confirmed for the standard FLUD [35]. When the FLUD-loaded liposomes, obtained through the extrusion and MVT methods, were incorporated into the culture medium (method 1), the FLUD inhibition activity on the *B. cinerea* conidial germination started from 0.3 µg·mL^−1^ of active compound, ranging from 6.7% (extruded plain) to 40% (MVT PEGylated) (Figure 4A). An exception was the extruded cationic liposomes, ineffective up to 0.3 µg·mL^−1^, which inhibited the germination of 92.3% of *B. cinerea* conidia at 1 µg·mL^−1^ FLUD (Figure 4A). The effectiveness of the FLUD-loaded liposomes in conidial germination inhibition was reduced by at least one hundred times as compared to the FLUD control and the extruded cationic liposomes tended to be more effective than the MVT cationic ones, while the PEG and plain MVT liposomes were more effective than the PEGylated and plain extruded ones.

Among the liposomes, although the plain formulation resulted in a greater release of the active substance at equilibrium, the MVT PEGylated allowed the best control, inhibiting conidial germination up to 40% when FLUD was applied at 0.3 µg·mL^−1^.

Plating the FLUD-loaded liposomes on the top of agar plugs (method 2), appreciable effectiveness was observed only at 1 µg·mL^−1^, ranging from 40% (MVT plain) to 100% (extruded cationic). However, no differences with respect to method 1 were observed for FLUD, and the MIC at 0.03 µg·mL^−1^ was confirmed (Figure 4B).

**Figure 5:**
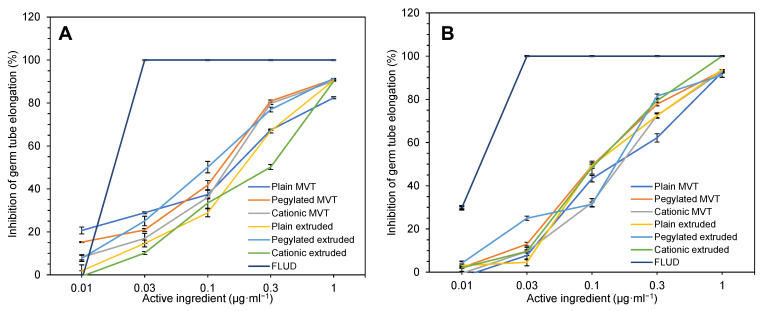
Inhibition of *B. cinerea* germ tube elongation following the application of different doses of FLUD or FLUD-loaded liposomes. (**A**) FLUD and FLUD-loaded liposomes were incorporated into the growth medium (method 1). (**B**) FLUD and FLUD-loaded liposomes were applied on the top of the medium (method 2). Bars indicated the standard error of three biological replicates.

A slight inhibition of germ tube elongation was observed when 0.01 µg·mL^−1^ of FLUD-loaded liposomes were added to the medium, and the effectiveness ranged from 1.9% (Extruded Plain) to 20.7% (MVT Plain). The active ingredient FLUD seems to be not effective on germ tube elongation at the lowest concentration useful for conidial germination (0.01 µg·mL^−1^). In this case, the better results were obtained by using MVT plain liposomes at the lowest doses applied (0.01 µg·mL^−1^), while the inhibitory activity of all the liposomes progressively and proportionally increased with the dose increase, reaching the higher reduction of germ tube elongation (90%) at the highest concentration assessed (1 µg·mL^−1^). Also, in this case, the technical grade FLUD allowed a total inhibition at 0.03 µg·mL^−1^ (Figure 5A).

A similar trend in terms of reduction of the germ tube elongation was observed in the assay with method 2 (Figure 5B), although lower effectiveness with respect to method 1 was recorded at 0.01 µg·mL^−1^. At the highest FLUD concentration assessed (1 µg·mL^−1^), the liposomes showed the highest biological activity, reducing the germ tube elongation from 91% (extruded PEGylated) up to 100% (extruded cationic).

**Figure 6:**
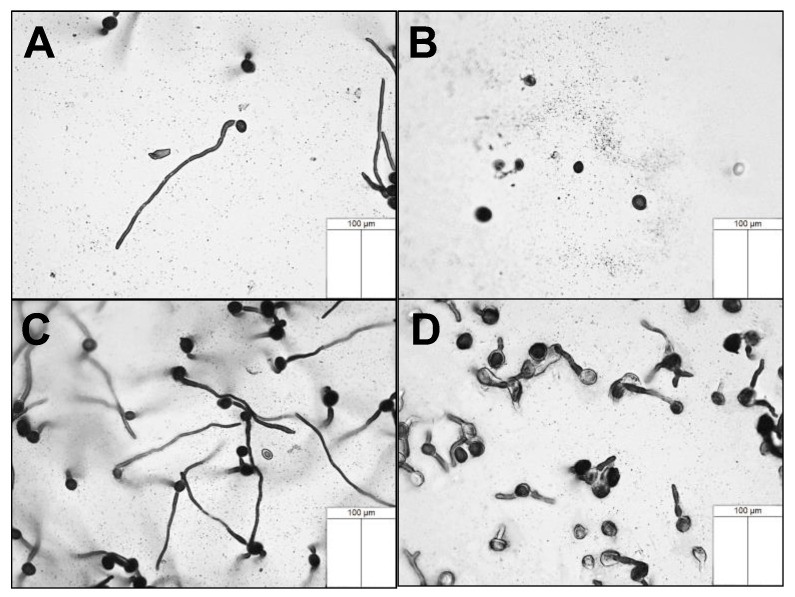
Germ tube length of conidia in (**A**) fungicide-unamended control medium (average length 108 µm)**,** (**B**) FLUD 0.03 µg·mL^−1^ (no germination), (**C**) Plain MVT control medium (average length 114 µm), (**D**) FLUD-loaded plain MVT liposomes at 1 µg·mL^−1^ (average length 20 µm).

When unloaded liposomes were evaluated, there was no significant difference in germ tube length compared to the control media (Figure 6).

**Figure 7:**
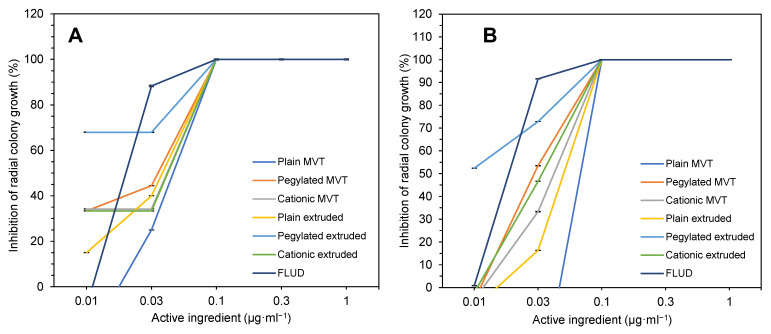
Inhibition of *B. cinerea* radial colony growth following the application of increasing doses of FLUD or FLUD-loaded liposomes. The orthogonal diameter of the colony was measured at (**A**) 3 and (**B**) 5 days after inoculation (DAI).

Greater effectiveness in the inhibition of colony growth, compared to FLUD, was generally confirmed three days after inoculation (DAI) at the lowest concentration assessed (0.01 µg·mL^−1^) with the FLUD-loaded liposomes, except for the MVT plain liposomes (Figure 7A). The extruded PEGylated liposomes showed the highest effectiveness, inhibiting radial colony growth up to 68%. For all the treatments, the MIC was 0.1 µg·mL^−1^.

At 5 DAI (Figure 7B), probably due to the better stability over time, only the extruded PEGylated liposomes still showed inhibitory activity at a FLUD concentration of 0.01 µg·mL^−1^, reducing the colony growth by 52%. Except for the MVT plain liposomes, having the lowest stability, all the liposomes showed good activity at 0.03 µg·mL^−1^, ranging from 16% (extruded plain) up to 72% (extruded PEGylated), while the control FLUD reduced the colony growth by 91%. For all the treatments, the MIC was confirmed at 0.1 µg·mL^−1^.

Extruded PEGylated liposomes appear to be the optimal compromise for active substance release. Following a slight control of conidial germination, although less effective than FLUD (22.7% at 0.3 µg·mL^−1^, in method 1), the formulation showed higher effectiveness than FLUD in the subsequent phases of germ tube elongation and mycelial growth (respectively, 7.7% and 68% at 0.01 µg·mL^−1^). These results agree with a slow release of FLUD by the liposome but prolonged over time, as also previously reported for calcium alginate nanocarriers and cypermethrin [36]. Consequently, although the application of FLUD-loaded liposomes was less effective than FLUD in conidial germination inhibition, the subsequent gradual and prolonged release of FLUD allowed better inhibitory activity against the subsequent stages of pathogen development. Furthermore, it is well known that *B. cinerea* produces different lytic enzymes, such as amylase, polygalacturonase, pectinase, and peroxidase, which are crucial for the infection process [37,38]. Following the germination, the release of lytic enzymes into the media could allow the degradation of the liposomal structure, determining the progressive release of the active compound. Consequently, the liposome formulations, and particularly the PEGylated extruded, mediating a slower release, could prevent the degradation of FLUD, making it available in the subsequent growth phases of the fungus, thus determining a higher effectiveness even at lower concentrations than the FLUD alone. Moreover, it is known the capacity of liposomes to adhere and fuse with negatively charged cell membranes, favoring the endocytosis of the conveyed compound [39,40]. The penetration capability of nanoparticles based on poly (lactic-co-glycolic acid) (PLGA NPs) in the hyphae of *B. cinerea*, enhancing the uptake and consequently the antifungal activity, is known [41]. This would explain the higher inhibition effectiveness on the mycelia growth of the cationic liposomes compared to the plain liposomes that showed poor efficacy at both 3 and 5 DAI.

## 3. Materials and Methods

### 3.1. Materials

Cholesterol, Didodecyldimethylammonium bromide (DDAB), the grade salts for phosphate-buffered saline solutions (PBS), potassium chloride, Sephadex G50 medium, dialysis tubes (12,400 Da), Tween20, and Dimethyl-sulfoxyde (DMSO) were obtained from Merck Italy (Merck Life Science S.r.l., Milan, Italy). The Lipoid S100 (LS100, ≥94.0% soybean phosphatidylcholine) was from Lipoid (Lipoid, Ludwigshafen, Germany). Polycarbonate membranes (porosity 100 nm), 1,2-dioleoyl-sn-glycero-3-phosphoethanolamine-N-(lissamine rhodamine B sulfonyl) (ammonium salt) (Liss Rhod PE) and 1,2-distearoyl-sn-glycero-3-phosphoethanolamine-N-[carboxy(polyethylene glycol)-2000] (sodium salt) (DSPE-PEG-2000) were purchased from Avanti Polar Lipids (Avanti Polar Lipids, Inc., Birmingham, AL, USA). Fludioxonil technical compound was provided by Syngenta Italy (Syngenta Italy, Milan, Italy). Malt extract was obtained from Oxoid (Oxoid Ltd., Basingstoke, United Kingdom), dextrose from Carlo Erba Reagents S.r.l (Carlo Erba reagents S.r.l., Cornaredo, Italy) and agar from LLG-Labware (LLG-Labware, Meckenheim, Germany). All the solvents used were LC-MS grade.

### 3.2. Liposome Preparation

Two methods were employed to prepare the liposomes, the so-called micelle-to-vesicle transition method (MVT) [42] and the extrusion method [43]. In both cases, three types of vesicles were prepared with the following compositions. Plain liposomes: 8 mg of LS100 and 10% (mol/mol) cholesterol; PEGylated liposomes: 8 mg of LS100, 10% (mol/mol) cholesterol, and 7% (mol/mol) of DSPE-PEG-2000; and cationic liposomes: 8 mg of LS100, 10% (mol/mol) cholesterol, and 25% (mol/mol) DDAB. The lipids were dissolved in chloroform and mixed thoroughly. Then, a thin lipid film was obtained after the complete evaporation of the organic phase under vacuum. For liposomes obtained with the MVT method, lipid films were hydrated with 0.5 mL of a sodium cholate solution at 4% (*w*/*w*) in PBS (pH 7.4) to obtain mixed micelles. The micellar suspension was subjected to size-exclusion chromatography (SEC) with Sephadex G50 medium to obtain 1 mL of liposomes. For liposomes obtained with the extrusion method, lipid films were hydrated with 1 mL of PBS. Then, the vesicle suspension size distribution was made uniform by the extrusion technique (mini-extruder Avanti Polar Lipids, 100 nm membranes). For drug-loaded liposomes, FLUD was added to the lipid blend in the proper concentration before obtaining the dried lipid film.

To obtain elution profiles of drug-loaded liposomes prepared by the MVT method, vesicles were fluorescently labeled by adding Liss Rhod PE (0.3% *w*/*w*) to the lipid blend before drying. Then, the mixed micelles (0.5 mL), obtained after dispersing the dried lipid film in the sodium cholate solution, were loaded into the SEC column (10 cm × 1 cm), and fractions of 0.25 or 0.5 mL were progressively collected. Each fraction was diluted 1:4 with PBS buffer and analyzed by UV-VIS spectroscopy and dynamic light scattering (DLS). In detail, the absorbance of the FLUD and Liss Rhod PE was monitored at 274 nm and 572 nm, respectively, while the DLS measurements were carried out at 25 °C and the light scattering intensity (dynamic) was registered. Normalized absorbances of FLUD and Liss Rhod PE and the light scattering intensity were then reported as a function of the elution volume.

### 3.3. Liposome Characterization

The liposomes were characterized from a dimensional point of view by measuring the hydrodynamic diameter and the polydispersity index (PDI) through DLS analysis with a Nanosizer ZS (Malvern Instruments, Malvern, UK). The Z potential was measured by laser Doppler electrophoresis with a Nanosizer ZS (Malvern Instruments, Malvern, UK). The FLUD content in the liposomes was evaluated spectroscopically upon drug extraction in a 1:1:1 ethanol/n-hexane/diethyl-ether mixture, as previously reported [44]. The drug signal was monitored at 274 nm with a Cary 5000 (Agilent Technologies, Santa Clara, CA, USA) ultraviolet-visible double-beam spectrophotometer. The results were used to calculate the percentages of encapsulation efficiency (EE%) and drug loading content (DLC%) of the FLUD into the liposomes as follows:

(1) $$EE\%=\frac{Mass_{encapsulated\;Fludioxonil}}{Mass_{total\;Fludioxonil\;added}}\times 100$$

(2) $$DLC\%=\frac{Mass_{encapsulated\;Fludioxonil}}{Mass_{liposomes}}\times 100$$

### 3.4. Liposome Stability

The stability of FLUD-loaded liposome formulations was investigated by incubating them in PBS at two different temperatures: 25 °C and 4 °C. The changes in initial particle hydrodynamic diameter were measured at different time intervals after a 1:10 dilution in PBS buffer by DLS analysis using a Dynamic Light Scattering Particle Size Analyzer LB-550 (HORIBA Ltd., Kyoto, Japan). Each experiment was performed in triplicate.

### 3.5. Fludioxonil Release Experiment

Cumulative in vitro drug release experiments were performed using a dialysis-based method. An amount of 3 mL of FLUD-loaded liposomes was diluted with 3 mL of phosphate buffer containing 30% (*V*/*V*) ethanol and then placed into dialysis tubes (12,400 Da) immersed in 100 mL of the same ethanol-containing buffer. Drug release was monitored throughout the experiment by collecting 1 mL of release medium at defined time intervals and replacing it with fresh buffer. The amount of released FLUD was monitored through absorbance spectroscopy. Cumulative drug release (*Q*%) was calculated according to the following equation:

(3) $$Q\left(\%\right)=\frac{C_{n}\times V_{t}+\sum_{i=1}^{n}C_{n_{i}-1}\times V_{a}}{Q_{t}}$$

where *Q* is the amount of released FLUD, *C_n_* is the drug concentration at a selected time point, *V_t_* is the total volume of medium, *V_a_* is the volume of the collected sample at each time point, and *Q_t_* is the initial amount of Fludioxonil in the liposomes.

### 3.6. Biological Assays

#### 3.6.1. Media

The doses were per liter of water, and all the media contained 20 g L^−1^ of agar. Malt extract agar (MEA: 20 g of malt extract, Oxoid) and potato dextrose agar (PDA: infusion of 200 g peeled and sliced potatoes kept at 60 °C for 1 h, 20 g of dextrose, adjusted at pH 6.5) were routinely used to grow the fungus. Dextrose agar (DA: 10 g of dextrose) was used for conidia germination, germ tube elongation assay, and for colony growth assay.

#### 3.6.2. Fungal Inhibition Assays

The biological activity of the FLUD-loaded liposomes was evaluated in vitro against *B. cinerea* reference strain SAS56 in terms of conidia germination, germ tube elongation and colony growth. The reference strain SAS56 is maintained in 10% glycerol solution at −80 °C in the culture collection of the Department of Soil, Plant and Food Sciences, University of Bari, as well as in the CBS collection (code number: CBS 145097), and routinely grown on MEA. The conidia used in the experiments were obtained on PDA as previously described [45], and two different methods were used to assess conidia germination. Five different concentrations (0.01; 0.03; 0.1; 0.3; 1 µg·mL^−1^) of FLUD active ingredient (Fludioxonil technical grade, Syngenta Crop Protection, Basel Switzerland) and in liposome formulations were incorporated into the DA medium, cooled down to 55 °C before plating (method 1), while the same amount of unloaded liposomes was added to the media as a control (unloaded control). DA disks (6 mm Ø) were placed on a glass microscope slide, inoculated with 10 µL of *B. cinerea* conidia suspension and incubated at 21 ± 1 °C in a humid chamber. In method 2, the *B. cinerea* conidia suspension was previously plated on the top of DA disks and, after adsorption, covered with 10 µL of FLUD or FLUD-loaded liposomes at the same concentrations tested with method 1. All the agar disks were maintained as above, and after 24 h, the conidia germination was stopped by adding a drop of lactophenol cotton blue stain. The percentage of germinated conidia showing normal germ tubes on the fungicide-amended media was assessed after 16–24 h of incubation at 21 ± 1 °C through observation under a microscope (DM2500, Leica Microsystems, Wetzlar, Germany, equipped with an ocular micrometer) (×200 magnification) of three random samples of 100 conidia per treatment. Fungicide-unamended DA was used as a control in all the experiments. The frequency of germinated conidia was calculated considering the frequency of conidial germination on the fungicide-unamended control medium. Three technical replicates were performed for each treatment.

For colony growth assay, three replicated Petri dishes (55 mm Ø) containing unamended DA or DA amended with five concentrations (0.01; 0.03; 0.1; 0.3; 1 µg·mL^−1^) of FLUD as the active ingredient or FLUD-loaded liposomes were inoculated with mycelium plugs (2 mm) from actively growing cultures on MEA. The plates were maintained at 21 ± 1 °C in the dark and two orthogonal diameters were measured 3 and 5 days after inoculation (DAI). At each condition, the inhibiting effect was calculated according to the Abbot Index [46]: (ULC-T)/ULC) × 100, where ULC is the value of conidial germination, germ tube elongation or mycelial growth on the control medium, and T is the value for the treatment.

#### 3.6.3. Statistics

One-way ANOVA and Tukey’s honest significant difference (HSD) were applied for statistical analysis of the data with CoStat software, version 6.451 (CoHort Software, Monterey, CA, USA).

## 4. Conclusions

Fludioxonil-loaded soy PC-derived liposomes by MVT or extrusion method were obtained for the first time and evaluated in preliminary application in vitro against the phytopathogenic fungus *B. cinerea*. Three different liposomes, plain, PEGylated, and cationic, were evaluated in terms of stability, release behavior and antifungal activity.

According to the data on release, the liposome structure behaves as a barrier, reducing the diffusion of FLUD, and compared to plain vesicles, the PEG coating confers further stabilization of the structure. This resulted in lower effectiveness than the FLUD on conidial germination; however, the liposomal formulation can improve the persistence and action period of the fungicide, extending the application interval and targeting various stages of pathogen development. Considering a release percentage of approximately 75% of the active compound at equilibrium, the PEG-coated liposomes appear to be more effective than the FLUD in the inhibition of germ tube elongation and radial colony growth, allowing a reduction in the use of active substances, while keeping or improving the effectiveness.

Although further in planta research should be conducted to investigate their real applicability to fighting fungal infections, liposomal formulations derived from soy PC are proving to be a promising tool to address the challenges of more efficient and sustainable agriculture.

## Figures and Tables

**Table 1:** Physicochemical and encapsulation properties of FLUD-loaded liposomes *.

	Micelle-To-Vesicle Transition Method				Extrusion Method			
	DLC	EE	Size	Z Potential	DLC	EE	Size	Z Potential
	%	%	nm	mV	%	%	nm	mV
Plain	0.38 ± 0.02	98 ± 5	36.4 ± 0.1	−8.7 ± 1.1	0.37 ± 0.02	98 ± 5	119.6 ± 0.6	−5.5 ± 1.1
PEGylated	0.29 ± 0.01	78 ± 3	31.4 ± 0.2	−6.5 ± 1.0	0.38 ± 0.02	100 ± 5	129 ± 1	−3.5 ± 1.0
Cationic	0.36 ± 0.02	96 ± 5	56.7 ± 0.4	+18.2 ± 1.7	0.34 ± 0.02	90 ± 4	127 ± 1	+36 ± 2

* The initial concentrations of FLUD and the lipids/FLUD molar ratio are 30 µg·mL^−1^ and 83.3, respectively. Results are presented as mean ± standard deviation (*n* = 3).

**Table 2:** Effectiveness of FLUD and FLUD-loaded liposomes on conidial germination and germ tube elongation of *Botrytis cinerea* was evaluated by incorporating the compounds in the growth medium (method 1) or applying them on the top of the medium after fungal inoculation (method 2). The data are mean values of percentage inhibition of conidial germination or germ tube elongation of three biological replicates and their standard errors.

Formulation (µg·mL^−1^)	Effectiveness on Conidial Germination					Effectiveness on Germ Tube Elongation				
	0.01	0.03	0.1	0.3	1	0.01	0.03	0.1	0.3	1
Method 1										
Plain MVT	0.0 ± 0.0	0.0 ± 0.0 ***	1.7 ± 0.3 ***	15.3 ± 1.8 ***	61.3 ± 3.6 ***	20.7 ± 1.6 ***	28.9 ± 0.5 ***	37.4 ± 2.0 ***	67.4 ± 0.2 ***	82.4 ± 0.5 ***
Pegylated MVT	0.0 ± 0.0	0.0 ± 0.0 ***	1 ± 0.5 ***	40 ± 0.8 ***	80.7 ± 1.9 ***	15.2 ± 0.2 ***	20.9 ± 0.6 ***	41.8 ± 2.1 ***	80.9 ± 0.5 ***	91.1 ± 0.0 ***
Cationic MVT	0.0 ± 0.0	0.0 ± 0.0 ***	0.3 ± 0.3 ***	27 ± 1.2 ***	76 ± 0.5 ***	8.1 ± 1.2	16.9 ± 2.4 ***	36.2 ± 0.7 ***	79.8 ± 0.5 ***	90.2 ± 0.0 ***
Plain extruded	0.0 ± 0.0	0.0 ± 0.0 ***	0.0 ± 0.0 ***	6.7 ± 0.3 ***	53.3 ± 1.4 ***	1.9 ± 2.7	14.6 ± 1.6 ***	28.9 ± 1.8 ***	66.9 ± 0.9 ***	90.5 ± 0.0 ***
Pegylated extruded	0.0 ± 0.0	0.0 ± 0.0 ***	0.0 ± 0.0 ***	22.7 ± 1.8 ***	59.7 ± 0.3 ***	7.7 ± 1.4	25.1 ± 2.2 ***	50.1 ± 2.7 ***	76.9 ± 1.1 ***	91.4 ± 0.0 ***
Cationic extruded	0.0 ± 0.0	0.0 ± 0.0 ***	0.0 ± 0.0 ***	0.0 ± 0.0 ***	92.3 ± 1.2	−0.7 ± 2.5	10.2 ± 0.7 ***	33.4 ± 2.4 ***	50.2 ± 1.2 ***	90.2 ± 0.0 ***
FLUD	0.0 ± 0.0	100 ± 0.0	100 ± 0.0	100 ± 0.0	100 ± 0.0	−2.4 ± 1.5	100 ± 0.0	100 ± 0.0	100 ± 0.0	100 ± 0.0
Method 2										
Plain MVT	0.0 ± 0.0	0.0 ± 0.0 ***	0.0 ± 0.0 ***	0.0 ± 0.0 ***	41 ± 3.1 ***	2.2 ± 4.8 ***	7.7 ± 1.9 ***	43.3 ± 1.5 ***	62.2 ± 1.9 ***	92.5 ± 0.0 ***
Pegylated MVT	0.0 ± 0.0	0.0 ± 0.0 ***	0.0 ± 0.0 ***	0.0 ± 0.0 ***	63.7 ± 3.1 ***	2.1 ± 1.7 ***	12.9 ± 0.8 ***	49.2 ± 0.5 ***	77.8 ± 0.8 ***	93.2 ± 0.0 ***
Cationic MVT	0.0 ± 0.0	0.0 ± 0.0 ***	0.0 ± 0.0 ***	0.0 ± 0.0 ***	90 ± 0.0	−0.5 ± 2.3 ***	9.6 ± 1.9 ***	32.1 ± 1.9 ***	72.5 ± 1.2 ***	92.9 ± 0.0 ***
Plain extruded	0.0 ± 0.0	0.0 ± 0.0 ***	0.0 ± 0.0 ***	0.0 ± 0.0 ***	62.3 ± 3.3 ***	3.1 ± 0.8 ***	4.6 ± 1.6 ***	49.3 ± 1.3 ***	72.4 ± 0.9 ***	93.8 ± 0.0 ***
Pegylated extruded	0.0 ± 0.0	0.0 ± 0.0 ***	0.0 ± 0.0 ***	0.0 ± 0.0 ***	90 ± 0.0	4.2 ± 3.2 **	24.9 ± 3.0 ***	31.4 ± 3.9 ***	81.5 ± 0.4 ***	91.2 ± 0.0 ***
Cationic extruded	0.0 ± 0.0	0.0 ± 0.0 ***	0.0 ± 0.0 ***	0.0 ± 0.0 ***	100 ± 0.0	2 ± 2.8 ***	9.6 ± 0.7 ***	48.4 ± 2.9 ***	79.4 ± 0.7 ***	100 ± 0.0
FLUD	0.0 ± 0.0	100 ± 0.0	100 ± 0.0	100 ± 0.0	100 ± 0.0	29.8 ± 0.7	100 ± 0.0	100 ± 0.0	100 ± 0.0	100 ± 0.0

Statistically significant differences from the FLUD were evaluated by one-way ANOVA followed by Tukey’s HSD test at significance levels: *p* ≤ 0.01 (**) and *p* ≤ 0.001 (***).

## Data Availability

Data available on request.
